# Characterization and phylogenetic analysis of the complete plastome of *Veronica undulata* (Plantaginaceae)

**DOI:** 10.1080/23802359.2021.1966345

**Published:** 2021-08-19

**Authors:** Kun Liu, Shou-Jin Fan

**Affiliations:** Shandong Provincial Key Laboratory of Plant Stress Research, College of Life Sciences, Shandong Normal University, Ji’nan, China

**Keywords:** *Veronica undulata*, plastome, phylogenomics

## Abstract

*Veronica undulata* is a perennial herb, and the complete chloroplast genome (plastome) of *V. undulata* was determined in this study. The results showed that the plastome size of *V. undulata* was 151,178 bp, including a large single-copy region (68,533 bp), a small single-copy region (21,403 bp), and two inverted repeat regions (25,566 bp). The total GC content of the plastome was 38.1%. We annotated 115 unique genes in the plastome, including 81 protein-coding genes (PCGs), 30 tRNAs, and four rRNAs. Phylogenetic analysis showed that the species of *V. undulata* and *Veronica* clustered together.

*Veronica undulata* is a perennial herb in the Plantaginaceae family (Albach and Chase 2001, 2004; Jensen et al. 2005). The species number in *Veronica* is very large, and many questions exist regarding the species classification of this genus (Albach et al. 2004). *V. undulata* was found on slopes flanking wetlands or ditches, which was widely distributed in China, Korea, Japan, Nepal, India, Pakistan, Afghanistan, Russian Federation, and the United States. As a large amphibious plant, *V. undulata* has important medicinal and ornamental value. Studies have shown that *V. undulata* was rich in iridoid glycosides (Taskova et al. 2010) and phenolic compounds (Li 1952; Boeger and Poulson 2003; Chen et al. 2003). *V. undulata* was previously reported to have cellular activity and anti-inflammatory activity (Saracoglu et al. 2011; Beara et al. 2015), which can help promote wound healing through hemostasis (Küpeli et al. 2005; Harput-Hudaverdi et al. 2008). In addition, the extraction of *V. undulata* also contains antioxidant, anticholinergic, and anticancer activities (Stojković et al. 2013; Sharifi-Rad et al. 2018). Therefore, *V. undulata* is considered a potential source of functional ingredients with a wide range of biological activities and holds great promise for pharmacological applications (Frontela-Saseta et al. 2013; He et al. 2015). In this study, the plastome of *V. undulata* was reported, which will provide a basic genetic resource for studying this important species and determining its phylogenetic position.

The fresh leaves of *V. undulata* were collected from the Lushan area of Shandong Province (36°21‘N, 118°4’E). The voucher specimens (20200709) of *V. undulata* were preserved at the College of Life Sciences, Shandong Normal University. The modified CTAB method was used for total plant DNA extraction (Doyle and Doyle 1987). Library preparation and paired-end sequencing work were completed on the Illumina Novaseq platform at Novogene (Beijing, China). Plastome was assembled with the Organelle Genome Assembler (OGA, https://github.com/quxiaojian/OGA). Plastome was annotated by the Plastid Genome Annotator (PGA, https://github.com/quxiaojian/PGA) (Qu et al. 2019) and manually corrected using Geneious v8.0.2 (Kearse et al. 2012). To further determine the phylogenetic position of *V. undulata*, the maximum likelihood (ML) tree was reconstructed using RAxML v8.2.10 with the 1000 rapid bootstrap replicates and the GTRGAMMA substitution model, using the alignment matrix of 115 unique genes generated by MAFFT v7.313 (Katoh and Standley 2013; Stamatakis 2014).

The complete plastome of *V. undulata* (GenBank accession number: MW783683) was 151,178 bp in length. The plastome was a quadripartite structure consisting of two single-copy regions separated by a pair of 21,539 bp inverted repeats. The large and small single-copy regions were 80,455 bp and 12,849 bp, respectively. The total GC content was 38.1%. A total of 115 unique genes were annotated in this plastome, including 81 protein-coding genes (PCGs), 30 tRNAs, and four rRNAs. Phylogenetic tree analysis showed that the species of *V. undulata* and *Veronica* clustered together (Figure 1).

**Figure 1.**
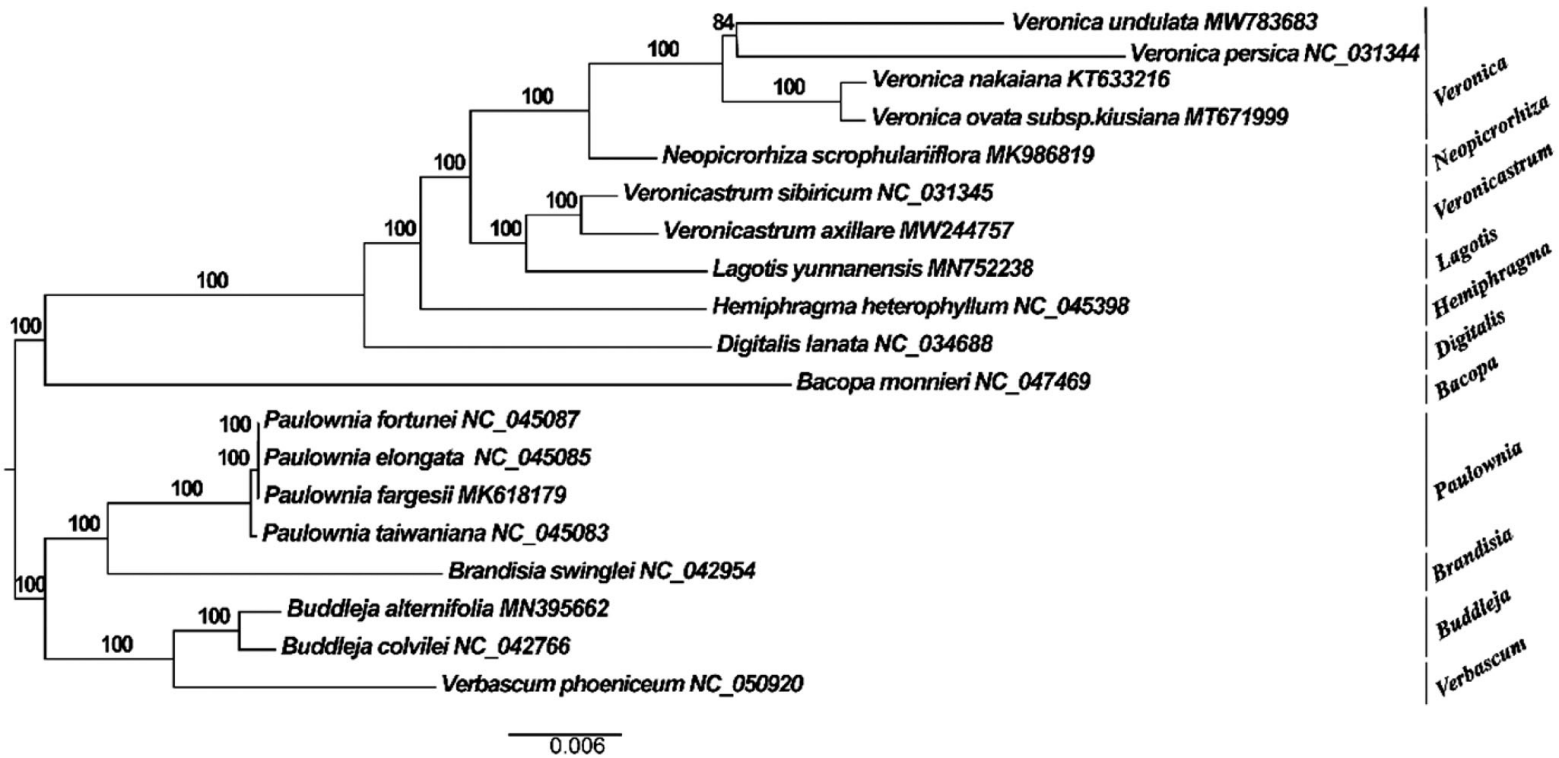
A maximum-likelihood (ML) tree inferred from 115 unique genes. *Paulownia*, *Brandisia*, *Buddleja* and *Verbascum* were used as outgroups. The numbers on branches are bootstrap support values.

## Data Availability

The genome sequence data that support the findings of this study are openly available in GenBank of NCBI at (https://www.ncbi.nlm.nih.gov/) under the accession no. MW783683. The associated BioProject, SRA, and Bio-Sample numbers are PRJNA720311, SRR14162363, and SAMN18646670, respectively.

## References

[CIT0001] Albach DC, Chase MW. 2001. Paraphyly of *Veronica* (Veroniceae; Scrophulariaceae): evidence from the internal transcribed spacer (ITS) sequences of nuclear ribosomal DNA. J Plant Res. 114(1):9–18.

[CIT0002] Albach DC, Chase MW. 2004. Incongruence in Veroniceae (Plantaginaceae): evidence from two plastid and a nuclear ribosomal DNA region. Mol Phylogenet Evol. 32(1):183–197. 15186806 10.1016/j.ympev.2003.12.001

[CIT0003] Albach DC, Martínez–Ortega MM, Fischer MA, Chase MW. 2004. A new classification of the tribe Veroniceae – problems and a possible solution. Taxon. 53(2):429–452.

[CIT0004] Beara I, Živković J, Lesjak M, Ristić J, Šavikin K, Maksimović Z, Janković T. 2015. Phenolic profile and anti-inflammatory activity of three *Veronica* species. Ind Crop Prod. 63:276–280.

[CIT0005] Boeger MRT, Poulson ME. 2003. Morphological adaptations and photosynthetic rates of amphibious *Veronica anagallis-aquatica* L. (Scrophulariaceae) under different flow regimes. Aquat Bot. 75(2):123–135.

[CIT0006] Chen X-X, Lang F-Y, Xu Z-H, He J-H, Ma Y. 2003. The occurrence of leafminers and their parasitoids on vegetables and weeds in Hangzhou area, Southeast China. BioControl. 48(5):515–527.

[CIT0007] Doyle JJ, Doyle JL. 1987. A rapid DNA isolation procedure for small quantities of fresh leaf tissue. Phytochemical Bulletin. 19(1):11–15.

[CIT0008] Frontela-Saseta C, Lopez-Nicolas R, Gonzalez-Bermudez CA, Martinez-Gracia C, Ros-Berruezo G. 2013. Anti-inflammatory properties of fruit juices enriched with pine bark extract in an *in vitro* model of inflamed human intestinal epithelium: the effect of gastrointestinal digestion. Food Chem Toxicol. 53:94–99. 23220608 10.1016/j.fct.2012.11.024

[CIT0009] Harput-Hudaverdi US, Oztunca FH, Saracoglu I. 2008. Comparative phytochemical and biological studies on *Veronica cuneifolia* subsp. *cuneifolia* and *V. cymbalaria*. Planta Med. 74(09):PC:88.

[CIT0010] He M-X, Hu Q-C, Zhu Q-L, Pan K, Li Q. 2015. The feasibility of using constructed wetlands plants to produce bioethanol. Environ Prog Sustainable Energy. 34(1):276–281.

[CIT0011] Jensen SR, Albach DC, Ohno T, Grayer RJ. 2005. *Veronica*: Iridoids and cornoside as chemosystematic markers. Biochem Syst Ecol. 33(10):1031–1047.

[CIT0012] Katoh K, Standley DM. 2013. MAFFT Multiple Sequence Alignment Software Version 7: Improvements in Performance and Usability. Mol Biol Evol. 30(4):772–780. 23329690 10.1093/molbev/mst010 PMC3603318

[CIT0013] Kearse M, Moir R, Wilson A, Stones-Havas S, Cheung M, Sturrock S, Buxton S, Cooper A, Markowitz S, Duran C, et al. 2012. Geneious Basic: an integrated and extendable desktop software platform for the organization and analysis of sequence data. Bioinformatics. 28(12):1647–1649. 22543367 10.1093/bioinformatics/bts199 PMC3371832

[CIT0014] Küpeli E, Harput US, Varel M, Yesilada E, Saracoglu I. 2005. Bioassay-guided isolation of iridoid glucosides with antinociceptive and anti-inflammatory activities from *Veronica anagallis-aquatica* L. J Ethnopharmacol. 102(2):170–176. 16019176 10.1016/j.jep.2005.05.042

[CIT0015] Li H-L. 1952. The Genus *Veronica* (Scrophulariaceae) in China. Proc Acad Nat Sci Phila. 104:197–218.

[CIT0016] Qu X-J, Moore MJ, Li D-Z, Yi T-S. 2019. PGA: a software package for rapid, accurate, and flexible batch annotation of plastomes. Plant Methods. 15(1):50. 31139240 10.1186/s13007-019-0435-7 PMC6528300

[CIT0017] Saracoglu I, Oztunca FH, Nagatsu A, Harput US. 2011. Iridoid content and biological activities of *Veronica cuneifolia* subsp. *cuneifolia* and *V. cymbalaria*. Pharm Biol. 49(11):1150–1157. 21595571 10.3109/13880209.2011.575790

[CIT0018] Sharifi-Rad J, Tayeboon GS, Niknam F, Sharifi-Rad M, Mohajeri M, Salehi B, Iriti M, Sharifi-Rad M. 2018. *Veronica persica* Poir. extract – antibacterial, antifungal and scolicidal activities, and inhibitory potential on acetylcholinesterase, tyrosinase, lipoxygenase and xanthine oxidase. Cell Mol Biol. 64(8):50–56. 29981683

[CIT0019] Stamatakis A. 2014. RAxML version 8: a tool for phylogenetic analysis and post-analysis of large phylogenies. Bioinformatics. 30(9):1312–1313. 24451623 10.1093/bioinformatics/btu033 PMC3998144

[CIT0020] Stojković DS, Živković J, Soković M, Glamočlija J, Ferreira ICFR, Janković T, Maksimović Z. 2013. Antibacterial activity of *Veronica montana* L. extract and of protocatechuic acid incorporated in a food system. Food Chem Toxicol. 55:209–213. 23333716 10.1016/j.fct.2013.01.005

[CIT0021] Taskova RM, Kokubun T, Ryan KG, Garnock-Jones PJ, Jensen SRJC, Bulletin P. 2010. Phenylethanoid and iridoid glycosides in the New Zealand snow hebes (*Veronica*, Plantaginaceae). Chem Pharm Bull. 58(5):703–711. 10.1248/cpb.58.703 20460800

